# Waves of Retrotransposon Expansion Remodel Genome Organization and CTCF Binding in Multiple Mammalian Lineages

**DOI:** 10.1016/j.cell.2011.11.058

**Published:** 2012-01-20

**Authors:** Dominic Schmidt, Petra C. Schwalie, Michael D. Wilson, Benoit Ballester, Ângela Gonçalves, Claudia Kutter, Gordon D. Brown, Aileen Marshall, Paul Flicek, Duncan T. Odom

**Affiliations:** 1 Cancer Research UK, Cambridge Research Institute, Li Ka Shing Centre, Robinson Way, Cambridge CB2 0RE, UK; 2 Department of Oncology, Hutchison/MRC Research Centre, University of Cambridge, Hills Road, Cambridge CB2 0XZ, UK; 3 European Bioinformatics Institute (EMBL-EBI), Wellcome Trust Genome Campus, Hinxton, Cambridge CB10 1SD, UK; 4 Wellcome Trust Sanger Institute, Wellcome Trust Genome Campus, Hinxton, Cambridge CB10 1SA, UK; 5 Cambridge Hepatobiliary Service, Addenbrooke's Hospital, Hills Road, Cambridge CB2 2QQ, UK

## Abstract

CTCF-binding locations represent regulatory sequences that are highly constrained over the course of evolution. To gain insight into how these DNA elements are conserved and spread through the genome, we defined the full spectrum of CTCF-binding sites, including a 33/34-mer motif, and identified over five thousand highly conserved, robust, and tissue-independent CTCF-binding locations by comparing ChIP-seq data from six mammals. Our data indicate that activation of retroelements has produced species-specific expansions of CTCF binding in rodents, dogs, and opossum, which often functionally serve as chromatin and transcriptional insulators. We discovered fossilized repeat elements flanking deeply conserved CTCF-binding regions, indicating that similar retrotransposon expansions occurred hundreds of millions of years ago. Repeat-driven dispersal of CTCF binding is a fundamental, ancient, and still highly active mechanism of genome evolution in mammalian lineages.

**PaperClip:**

## Graphical abstract

**Graphical Abstract:**

**Figure:**
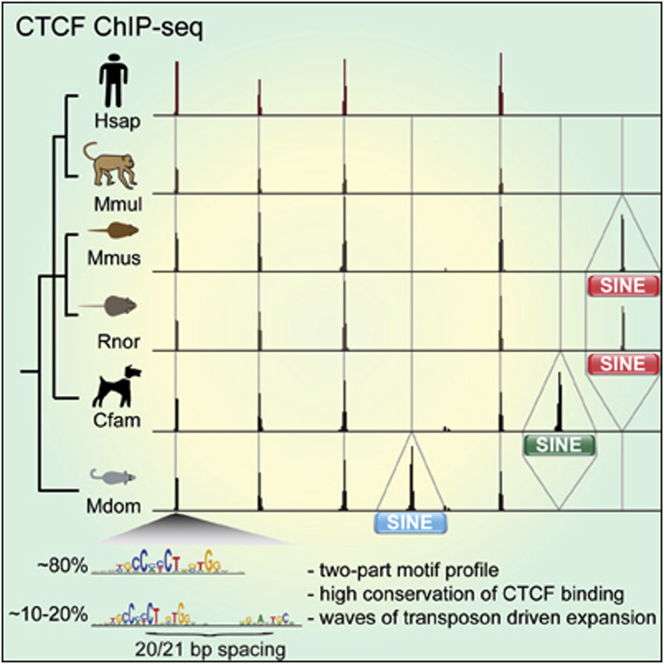

**Highlights:**

► CTCF-binding locations are highly conserved across mammals ► New locations for CTCF binding are carried by SINE repeats in many mammals ► Ancient and newly born CTCF-binding events similarly demarcate chromatin barriers ► Retroelements can reposition organizing elements throughout the genome

## Teaser

Evolutionary analysis of six divergent mammals uncovers both highly conserved and species-specific binding locations of the chromatin organizer, CTCF. This study reveals that activation of retroelements drives genome evolution by repositioning genome-organizing elements.

## Introduction

In contrast to exons and structural RNA sequences, genomic regions bound by proteins such as transcription factors (TFs) can change rapidly in mammalian genomes. One apparent exception may be the sequences bound by CCCTC-binding factor (CTCF), a DNA-binding protein that can divide transcriptional and chromatin domains, help direct the location of cohesin, and orchestrate global enhancer-promoter looping (for reviews, see Dunn and Davie, 2003; Phillips and Corces, 2009). CTCF is an essential (Fedoriw et al., 2004; Heath et al., 2008; Splinter et al., 2006), widely expressed nuclear protein with an 11 zinc finger DNA-binding domain that is highly conserved from fly to human (Burcin et al., 1997; Klenova et al., 1993; Moon et al., 2005). Originally identified as a transcriptional regulator for the *c-myc* oncogene (Baniahmad et al., 1990; Filippova et al., 1996; Lobanenkov et al., 1990), CTCF remains the only identified sequence-specific DNA-binding protein that helps establish vertebrate insulators (Bell et al., 1999). Additionally, CTCF has been implicated in transcriptional activation, repression, silencing, and imprinting of genes (Awad et al., 1999; Burcin et al., 1997; Filippova et al., 1996; Klenova et al., 1993; Lobanenkov et al., 1990).

Despite its importance to mammalian genome function and regulation, different preferred binding sequences for CTCF have been reported. A 15 to 20 bp core consensus sequence represented in nearly all CTCF-binding events was identified using genome-wide chromatin immunoprecipitation (ChIP) data (Kim et al., 2007). Subsequent studies have confirmed this result in different mouse, human, and chicken cells (Chen et al., 2008; Cuddapah et al., 2009; Heintzman et al., 2009; Jothi et al., 2008; Schmidt et al., 2010a). Earlier studies suggested that different combinations of zinc fingers might target sequences with lengths varying between 20 and 40 bp (Filippova et al., 1996; Ohlsson et al., 2001). Indeed, the DNase I footprint of CTCF at the *amyloid precursor protein* (*APP*) promoter is 40 bp in length (Quitschke et al., 2000). The apparent disconnect between in vivo binding specificity and the observed in vitro binding preferences has yet to be fully resolved (see Phillips and Corces, 2009).

How do CTCF-binding sequences change and emerge? The sequences bound by TFs evolve rapidly, most likely the result of genetic drift (ENCODE Project Consortium, 2007; Borneman et al., 2007; Dermitzakis and Clark, 2002; Kunarso et al., 2010; Odom et al., 2007; Schmidt et al., 2010b), whereas large, information-rich motifs, such as the one bound by CTCF, are likely to be selectively conserved. For example, CTCF's multiple roles, including division of chromatin and gene expression domains, have been reported to place strong purifying evolutionary pressure on bound regions (Kim et al., 2007; Xie et al., 2007; Bourque et al., 2008; Kunarso et al., 2010; Martin et al., 2011; Mikkelsen et al., 2010). Despite this likely selective pressure, evidence suggests that CTCF binding is also evolving rapidly, most notably the discovery that mouse embryonic stem cells (ESCs) have thousands of CTCF-binding events that cannot be conserved in the human genome, as they are found in rodent-specific B2 repeat elements (Bourque et al., 2008). This is consistent with early models (McClintock, 1950) and recent examples of repetitive elements driving regulatory divergence in eukaryotic genomes (Bejerano et al., 2006; Bourque et al., 2008; Britten, 1997; Han et al., 2004; Kunarso et al., 2010; Markljung et al., 2009; Mikkelsen et al., 2010; Wang et al., 2007; Lynch et al., 2011).

By analyzing the evolution of CTCF binding in six representative mammals, we found that CTCF binds a 33/34 bp motif with a two-part profile that is conserved across mammals, providing an explanation for the observed CTCF target sequence discrepancies. Remarkably, the bound sequences exhibit a hierarchy conserved across mammals, wherein frequently used motif instances underlie CTCF-binding events that are both most conserved and most resilient to changes in nuclear concentration of CTCF after RNAi knockdown. Moreover, in most species examined, we found that CTCF-binding events are associated with repeat element expansions, revealing the mechanism by which they are born. Functional studies illustrate that both newborn and conserved CTCF-binding events act as chromatin and gene expression barriers with similar frequency. Together, our results support a repeat-driven mechanism for functional CTCF-binding expansion, which is currently active in multiple mammals and was active in our common ancestor, thus creating the CTCF-binding events shared across mammals.

## Results

### CTCF-Binding Events Are Markedly More Conserved among Mammals than Tissue-Specific TF Binding in Mammalian Genomes

**Figure S1:**
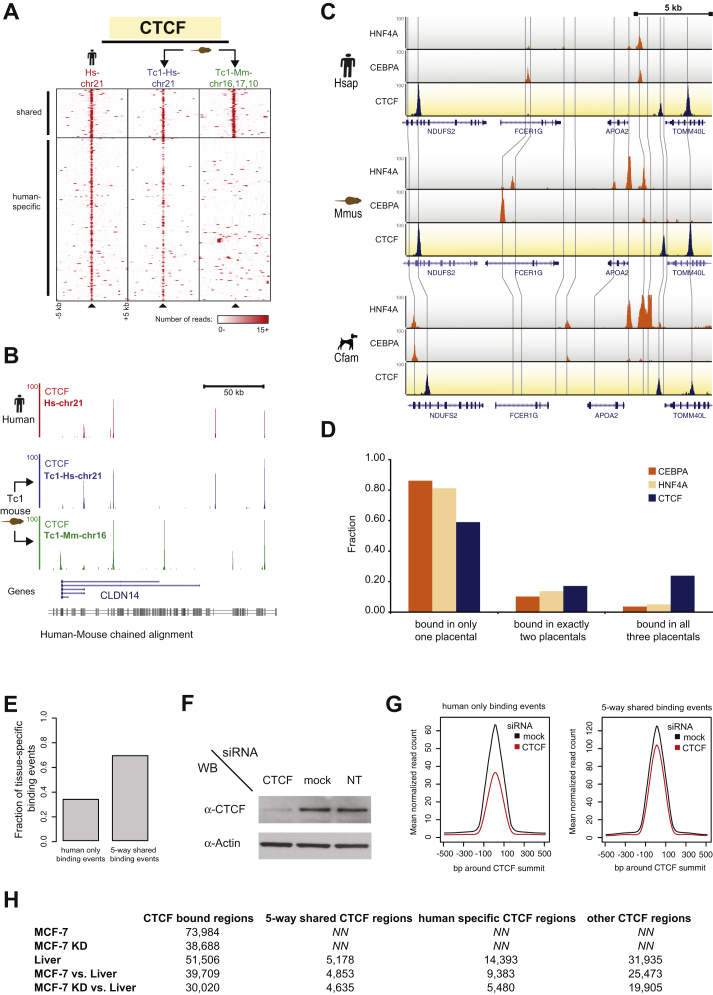
CTCF Binding Is Primarily Directed by Genetic Sequence and Is Highly Conserved; Western Blot Confirmation of CTCF RNAi and Tissue Specificity of Conserved CTCF Binding, Related to Figure 1 (A) In the first column (Hs-chr21), ten kilobase windows around human CTCF-binding events were ordered, based whether a syntenic CTCF-binding event is present or not in mouse liver. In the second and third columns, CTCF binding in the Tc1 mouse has been shown for the human chromosome 21 (Tc1-Hs-chr21) and for the orthologous mouse sequences (Tc1-Mm-chr16, 17, 10). Most CTCF binding found on human chromosome 21 in human liver is recapitulated in the mouse liver. (B) Genome tracks displaying the CTCF binding found near the liver-expressed gene CLDN14 in human (red, Hs-chr21) and Tc1 mouse (blue, Tc1-Hs-chr21; green, Tc1-Mm-chr16). (C) Genomic occupancy of HNF4A, CEBPA (orange tracks), and CTCF (blue tracks) is shown around the liver gene APOA2 in human, mouse, and dog. Grey lines connect orthologous regions between species. (D) Binding events for CEBPA, HNF4A, and CTCF have been sorted, based on whether they occur in one, two, or three of the placental species from (C). (E) The fraction of binding events found only in human (human only) or shared among all placental mammals (five-way) were characterized by their tissue specificity. Few deeply shared CTCF-binding events are tissue specific in humans. (F) Western blot of nuclear extracts after CTCF RNAi, mock RNAi, and non-transfected (NT) human MCF-7 cells. (G) Read profiles of CTCF binding after CTCF (red lines) or mock RNAi (black lines) in MCF-7 cells. CTCF binding was separated into two groups: (1) human-specific binding events in liver that are also found in MCF-7 cells (human only binding events) and (2) five-way shared binding events in liver that overlap with CTCF binding in MCF-7 cells (five-way shared binding events). (H) The total numbers of CTCF-binding events (CTCF-bound regions) for the following data sets are shown: MCF-7 after mock RNAi (MCF-7), MCF-7 after CTCF RNAi (MCF-7 KD), human liver (Liver). The bottom two rows show the CTCF binding overlaps between MCF-7 versus Liver and MCF-7 KD versus Liver binding. Total CTCF-binding overlaps are indicated on the left and further split into three categories: five-way shared, human-specific, and all other CTCF binding.

We used ChIP followed by sequencing (Table S1 available online) to determine CTCF binding in livers isolated from five eutherian mammals (human, macaque, mouse, rat, and dog) and the metatherian gray short-tailed opossum and confirmed that CTCF binding is mainly directed by genetic sequence rather than nuclear environment (Wilson et al., 2008) (Figures S1A and S1B).

We first compared CTCF-binding conservation with matched genome-wide data available for the TFs HNF4A and CEBPA in mouse, dog, and human (Schmidt et al., 2010b). Consistent with prior reports (Kunarso et al., 2010), we observed substantially higher conservation among CTCF-binding events than among liver-specific TFs, even near direct liver-specific target genes. For example, HNF4A and CEBPA binding has extensively diverged around the CEBPA target gene *APOA2* (Schmidt et al., 2010b), yet the CTCF-binding events in the same region are uniformly conserved in all three mammals (Figure S1C). Globally, CTCF binding is shared five times as often among human, dog, and mouse, as are CEBPA and HNF4A; conversely, CTCF has proportionally less lineage-specific binding (Figure S1D).

**Figure 1:**
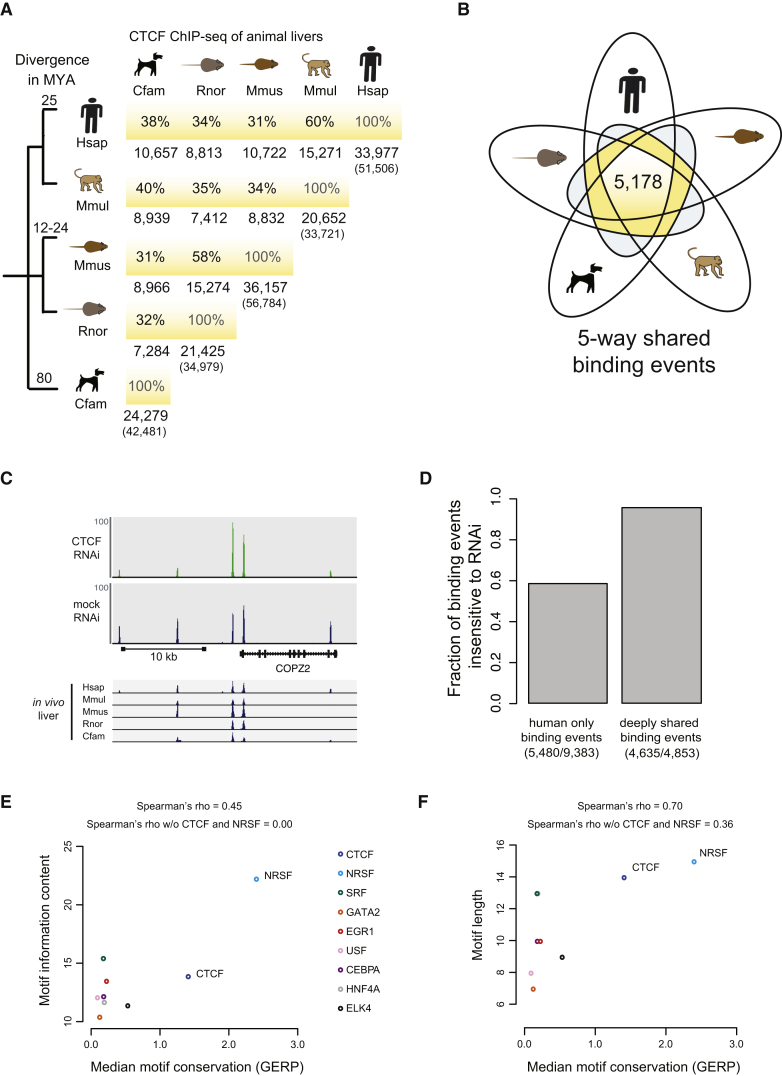
CTCF Occupancy in Five Placental Mammalian Genomes Reveals a Large Core Set of Conserved Binding (A) The total numbers of CTCF-binding events found in orthologous locations between each pair of placental species are shown as row-column intersections. The right-most numbers for each species represent all alignable CTCF-binding peaks (total peaks are in parentheses). Percentages are percentage-averages between pairwise species (Experimental Procedures). (B) Five-way comparison of CTCF binding in five placental mammals identified a shared set of 5,178 CTCF-binding events. (C) The upper track shows CTCF binding after CTCF knockdown (CTCF) in human MCF-7 cells (Figure S1F). The track immediately below shows CTCF binding with control RNAi (mock). The bottom five tracks show CTCF-binding data in liver of five mammalian species in syntenic regions, demonstrating that highly conserved CTCF-binding events are less sensitive to perturbation by RNAi knockdown. (D) The fraction of binding events found only in human (human only) or shared among all placental (five-way) were characterized by their sensitivity to RNAi knockdown of CTCF protein. Very few deeply shared CTCF-binding events were affected by CTCF knockdown. (E) Relation between motif information content and motif sequence conservation for nine TFs in human. (F) Relation between motif length and motif sequence conservation for the same TFs as in (E). See also Figure S1 and Table S2.

The inclusion of rat and macaque allowed us to compare closely related species, which overlapped by up to 60% in shared CTCF binding. In fact, as might be expected, CTCF-binding divergence generally corresponded with evolutionary distance (Figure 1A).

More importantly, we observed a core set of over 5,000 CTCF-binding events shared by all five eutherian mammals (Figure 1B) and found across numerous human tissues (Figure S1E). Conserved CTCF-binding events are less sensitive than species-specific binding events to reduced levels of the CTCF protein. We analyzed CTCF binding before and after RNAi knockdown in human MCF-7 cells (Schmidt et al., 2010a) (Figures 1C, S1F, and Extended Experimental Procedures) and found that virtually all binding events conserved across five species were resistant to knockdown, compared to only 60% of human-specific binding events (Figure 1D). Thus, conserved binding events are highly stable protein-DNA interactions, suggesting that they play functional roles in many cell types.

Although higher conservation among CTCF-binding events, relative to tissue-specific TFs, could be due solely to the information content or length of the bound motif, we found that conservation of CTCF-binding events across mammalian genomes is not purely the result of a longer target motif. We observed an increase in shared binding events between human, mouse, and dog with motif lengths from CEBPA (4%), to HNF4A (5%), to CTCF (24%), but we did not see a significant dependence when looking at a collection of sequence-specific factors. The median sequence conservation of a cross-section of motifs (ENCODE Project Consortium, 2011) revealed some degree of correlation with the motifs' length and information content; however this was not statistically significant and was largely due to the inclusion of CTCF and NRSF/REST (see Extended Experimental Procedures and Figures 1E and 1F). After excluding CTCF and NRSF, the other TFs showed very little to no correlation. Together, these data show that over 5,000 CTCF-binding events are highly biochemically and evolutionarily stable across mammalian species.

### CTCF Binds a DNA Motif Containing a Two-Part Profile

**Figure 2:**
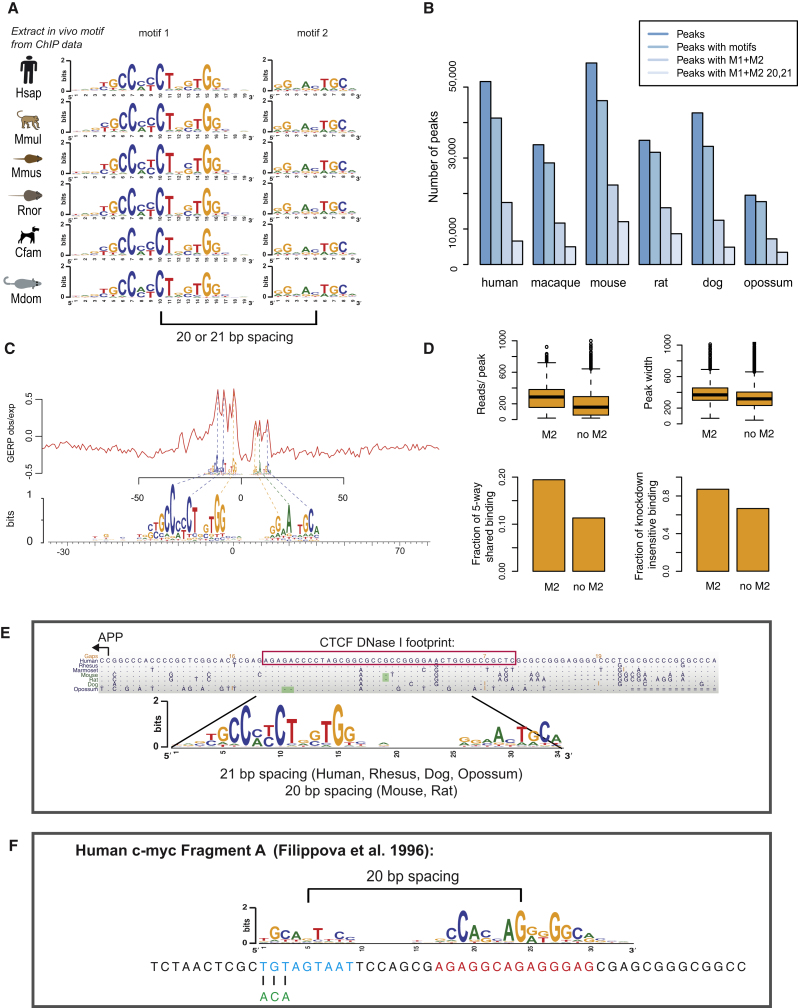
CTCF Binding Often Occurs at a Highly Conserved Motif, Consisting of a Two-Part Profile (A) Motifs (M1 and M2) identified de novo from CTCF-binding events. (B) Binding event counts and number of binding events with at least one motif (M1 and M1+M2) in all six species. M1+M2 20,21 represents the preferred spacing patterns of these two submotifs. (C) The DNA sequence constraint around the CTCF motif in human was plotted by observed/expected genomic evolutionary rate profiling (red, GERP) scores (Cooper et al., 2005). The frequencies of unchanged bases in five-way shared CTCF-binding events are shown as position weight matrix (PWM) below the GERP profile. (D) Peaks containing the M2 motif in preferred spacing are stronger in ChIP enrichment both by read count and peak width, are more highly shared among mammals, and are resistant to RNAi-mediated knockdown. (E) A multiple mammalian sequence alignment of a CTCF peak at the APP gene is shown. The DNase I footprint (red box, Quitschke et al., 2000) encompasses a complete 34 bp M1 and M2 CTCF motif. (F) DNA sequence of the human c-*myc* promoter (Human c-*myc* Fragment A) bound by CTCF in vivo and in vitro (Filippova et al., 1996*)*. The sequence contains the canonical M1 CTCF motif (red) and the M2 motif (blue). A 3 bp mutation in the M2 motif that eliminates CTCF binding in vitro is indicated in green. See also Figure S2.

**Figure S2:**
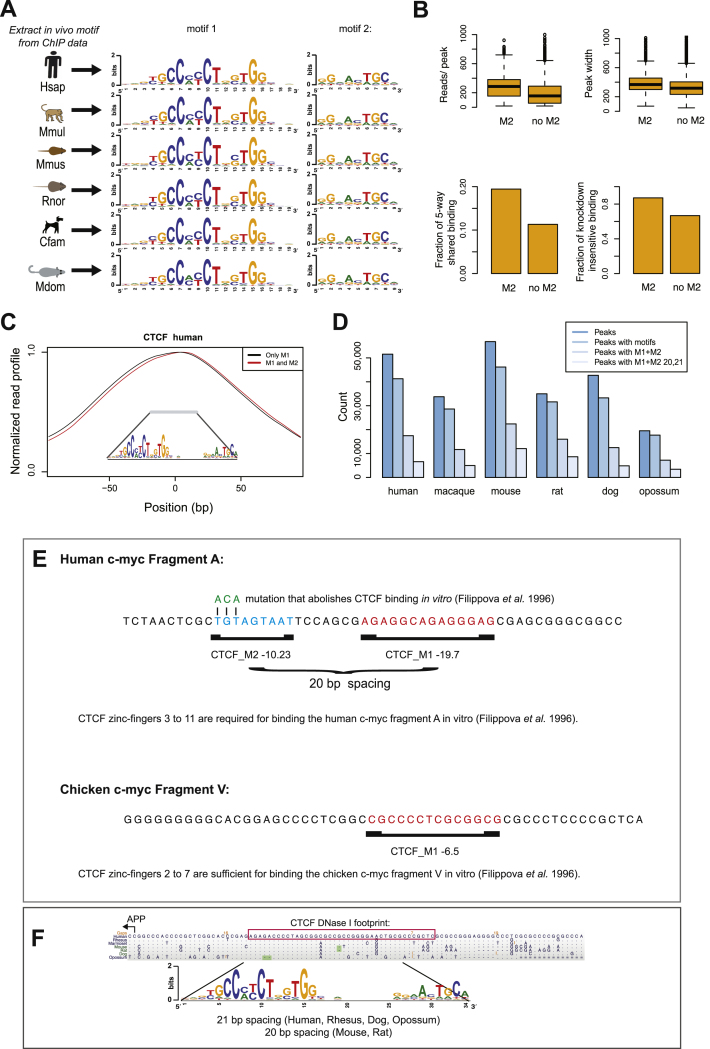
CTCF Motifs (M1 and M2) and Motif Occurrences, Related to Figure 2 (A) Motifs identified de novo from CTCF-binding events in all six species. (B) Different properties of CTCF-binding events dependent on the presence of M2. (C) Read profile at CTCF-binding events where only the M1 motif (black line) or the complete two-part motif consisting of M1 and M2 was detected (red line). (D) Binding event counts and number of binding events with at least one motif (M1 and M1+M2) in all six species. (E) Presence and absence of M1 and M2 in two DNA sequences from Filippova et al. (1996). The motif score (nmscan uses bits-suboptimal scoring with 0.0 being a perfect match) is indicated under each motif instance. (F) A multiple mammalian sequence alignment of a CTCF peak at the APP gene is shown. The DNase I footprint (red box, Quitschke et al., 2000) encompasses a complete 34 bp M1 and M2 CTCF motif.

Our genome-wide data for CTCF binding in livers of five eutherian species allowed us to identify de novo DNA sequences associated with CTCF binding at hundreds of thousands of locations. In addition to the known 20 bp motif, we further discovered a second 9 bp motif present at high frequency and with consistent spacing in each species. Both halves of the motif are unchanged across 180 million years of evolution, consistent with the high conservation of CTCF's DNA-binding domain (Figure S2), and create together a two-part 33/34 bp binding motif, which occurs in a quarter to a third of CTCF-binding events (Figures 2A and 2B). The second motif is downstream by either 21 or 22 bp from the center of the previously identified motif in approximately equal proportions in all studied species, except mouse and rat (Figure 4). Henceforth, we will refer to the canonical 20 base motif as M1 and to the 9 base motif as M2. The M2 motif has previously been found in CTCF DNase footprints, but the role of this motif is unknown (Boyle et al., 2011). The variable presence of the shorter and less information-rich M2 agrees with earlier suggestions that CTCF may have multiple binding modalities (Burcin et al., 1997; Filippova et al., 1996).

To characterize the importance of M2 for CTCF binding, we first identified binding events conserved in five placental mammals that contain both M1 and M2. Then we searched for evidence of positional sequence conservation of the M2 submotif. Plotting the frequency of all unchanged bases in the multiple species alignment revealed that the bases associated with both M1 and M2 were much less likely to see sequence changes compared to both the spacer and surrounding sequences where background levels are observed (Figures 2 and S2). We used genomic evolutionary rate profiling (GERP), a specific model of evolutionary constraint at the sequence level, to confirm this observation of purifying selection on both the previously known and the newfound motif bases (Cooper et al., 2005) (Figure 2C).

We found that binding events containing the full 33/34 bp motif show stronger ChIP enrichment, are more conserved, and remain less sensitive to CTCF knockdown compared to binding events containing only the M1 motif (Figure 2D). Moreover, the CTCF-binding peak is offset from the center of the M1 motif, consistent with CTCF binding to a larger, directional 33/34 bp motif. In cases where the M2 motif is present, this effect is slightly stronger (Figure S2C). These results indicate that CTCF directly binds M1+M2 in a highly conserved manner (Figure S2D).

### Hierarchical Motif-Word Usage of CTCF Is Conserved among Mammals

**Figure 3:**
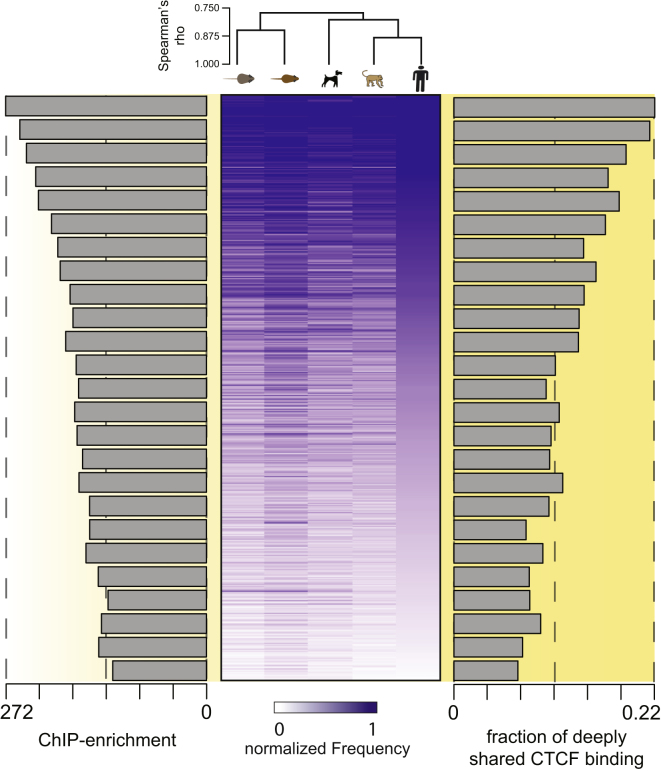
CTCF Motif Usage Shows a Conserved Hierarchy among Placental Mammals Heat map of the 2,492 CTCF motif-words found at least five times in any species anchored to human; words are normalized by their background occurrences within each genome. This set of words is found in 27,543 human-binding events. The data are sorted in the human column by decreasing frequency, and spearman rank correlations after one-dimensional hierarchical clustering of the rows are shown. The average ChIP-enrichment of the motif-words separated into bins containing 100 words is shown as a bar chart (left). Similarly, the fraction of five-way conserved CTCF-binding events within the same bins are shown as a bar chart (right). See also Figure S3.

**Figure S3:**
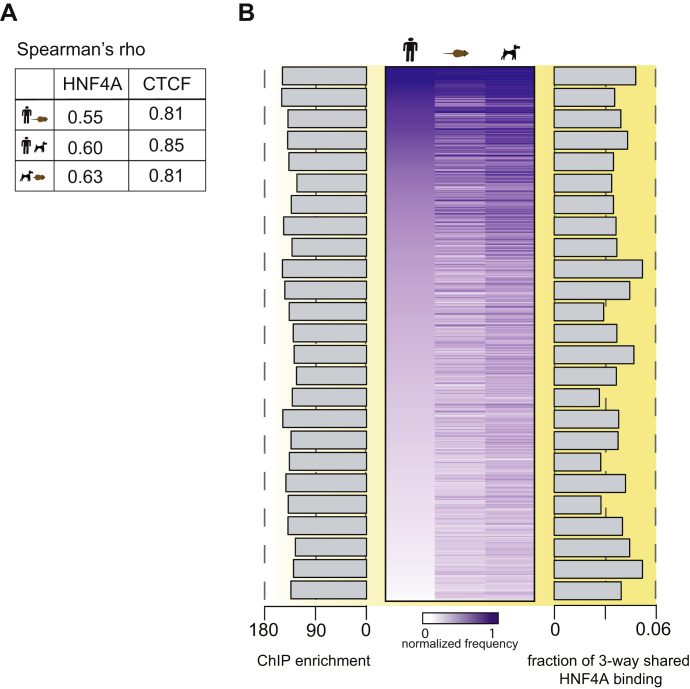
Motif-Word Analysis for HNF4A, Related to Figure 3 (A) Spearman correlations of HNF4A and CTCF motif-word usage between the indicated species pairs. (B) Heat map of 3,981 HNF4A motif-words found at least five times in any species; words are normalized by their background occurrences within each genome. This set of words is found in 17,661 human binding events. The data are sorted in the human column by decreasing frequency. The average ChIP-enrichment of the HNF4A motif words separated into bins containing 100 words is shown as a bar chart (left). Similarly, the fraction of three-way conserved HNF4A binding events within the same bins are shown as a bar chart (right).

The position weight matrix of CTCF's binding motif is composed of thousands of specific sequences, or motif-words. We tested whether CTCF has a preferred set of motif-words by analyzing their frequency of occurrence. We clustered highly similar motif-words using the 14 most informative bases of the M1 motif, which together capture over 95% of the motif's information content. A set of 33,994 different 14-mer motif-words (out of a possible 69,865) are used by CTCF at least once in the five placental mammals. We found that a small subset of these tens of thousands of motif-words are disproportionately often bound by CTCF within and between different species (Figure 3). For example, the top 200 bound motif-words are responsible for 4,006 binding events in the human genome; in fact, just 2,492 words (3.6% of the possible words) account for over half of the binding events in the human genome. CTCF motif-word usage is strikingly conserved between the species (Spearman rank correlation > 0.76) and recapitulates both the evolutionary distances of the species as well as key characteristics of the CTCF-binding events (Figure 3). In particular, we observed that the frequency of a word's usage positively correlates with both the likelihood of a binding event being shared among all five species and the strength of the ChIP enrichment (Figure 3). A similar analysis for a typical tissue-specific TF (HNF4A) showed considerably lower correlation of motif-word usage (Figure S3A) and no correlation between word frequency and either conservation or ChIP enrichment (Figure S3B). Collectively, these results reveal a functional hierarchy of CTCF-bound motif-words maintained during evolution.

### Lineage-Specific Repeats Drive Divergence of CTCF Binding in Many Mammalian Lineages

The existence of a motif-word usage hierarchy as well as thousands of highly conserved CTCF-binding events is inconsistent with prior models proposing rapid TF birth by neutral mutation and natural selection (MacArthur and Brookfield, 2004).

**Figure 4:**
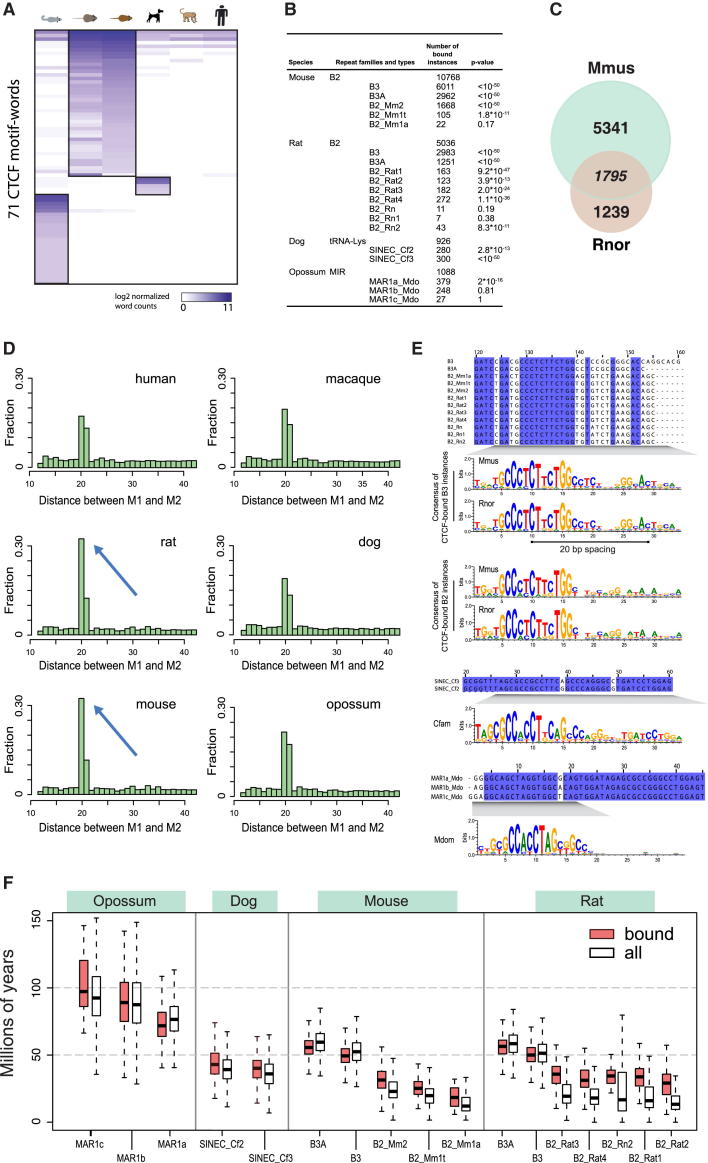
Repeat Expansions Remodeled CTCF Binding in Three Mammalian Lineages (A) Heatmap of 71 motif-words identified as highly enriched in mammalian lineages. (B) Lineage-specific repeats that are associated with the lineage-specific motif-words. (C) Venn diagram showing the number of B2 repeat-associated binding events shared between mouse and rat. (D) Frequencies of distances between the centers of M1 and M2 in all six studied species. There is a smaller spacing between M1 and M2 in mouse and rat (blue arrow), due to the B2 repeat expansion. (E) Sections of the aligned consensus sequences from CTCF-carrying retrotransposons in mouse, rat, dog, and opossum; rat and mouse contain the M1+M2 motif, dog and opossum only contain M1. Consensus motifs for CTCF binding solely based on bound repeat instances are shown below each alignment. (F) Estimated ages of lineage-specific repeats that expanded CTCF binding. White box plots are all instances of the indicated repeat; red box plots are only those bound by CTCF. See also Figure S4.

**Figure S4:**
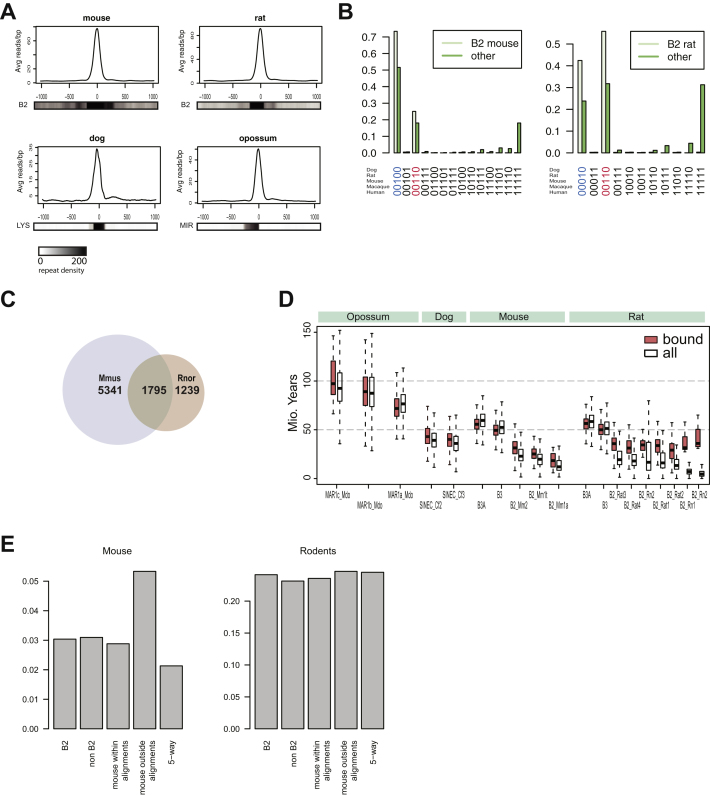
CTCF Directly Binds Specific Repeat Elements, and Mouse and Rat Share Many Bound B2 Repeat Instances, Related to Figure 4 (A) Aggregate read-profiles of repeat driven CTCF-binding events in four mammals. The bars under the graphs show the density of the indicated repeats. (B) The fraction of CTCF-binding events due to B2 repeats and all other binding events in mouse (left) and rat (right) are separated into different conservation groups. A “1” indicates binding, and “0” indicates no binding in the relevant species. For example binding events that are only shared between mouse and rat are depicted as “00110” and also highlighted in red. More than half of the B2 repeats bound by CTCF in rat are also bound in mouse, indicating that the SINE transposon acquired CTCF binding in a common ancestor of rat and mouse. (C) Venn diagram showing the number of B2 repeats associated binding events in the alignable genome shared in mouse and rat. (D) Estimated ages of lineage-specific repeats that expanded CTCF binding. The white box plots are based on all instances of the indicated repeat; the red box plots are only based on repeat instances that are bound by CTCF. (E) Fraction of different CTCF-binding event categories associated with mouse (Mouse) or rodent (Rodents) specific genes.

We therefore searched for an alternative mechanism for the de novo creation in a common mammalian ancestor of the thousands of CTCF-binding events now found throughout mammals. Despite the generally high conservation of CTCF motif-word usage, we noted that specific sets of motif-words were overrepresented in rodents (mouse and rat), dog, and opossum (Figure 4A). We found that the vast majority of these overrepresented motif-words are embedded within SINE transposons (Figures 4B and S4).

In mouse, this observation is consistent with previous reports showing that the CTCF motif was carried to over 10,000 locations in the mouse genome by the B2 SINE family (Bourque et al., 2008), which has expanded significantly in rodents (Kass et al., 1997). We further discovered that CTCF binding has been spread to thousands of locations in the rat genome, also via muridae-specific B2 SINEs (Figure 4B). About 2,000 binding events found within B2 elements are shared by mouse and rat, whereas approximately 5,300 B2-associated binding events are found uniquely within mouse and over 1,200 solely in rat (Figures 4C and S4). Thus, the B2 expansion was active before the speciation of rats and mice and remained active in both lineages after speciation. The thousands of rodent-expanded B2-associated CTCF-binding events, most of which contain a full 33 bp CTCF motif with a 20 bp spacing between M1 and M2, have profoundly influenced the occurrence of specific bound CTCF motifs (Figure 4D). However, the B2-associated CTCF-binding events seem not to be enriched near mouse- or rodent-specific genes compared to other binding events (Figure S4).

Similarly, we found that the SINEC-Cf member of the canoidea-specific SINE repeat family LYS has carried CTCF-binding events through the dog genome (Figure 4). In contrast to rodents, the dog-specific expansion appears more limited, resulting in well under a thousand binding events and a sequence that is centered solely on the M1 motif (Figure 4E).

Similar to rodents and dogs, word-level analysis of the CTCF-binding events revealed a set of motif-words overrepresented in opossum, frequently associated with opossum-specific SINE repeats MAR_Mdo (MIR family) (Figures 4A and 4B). Opossum is the closest out-group to the eutherian mammals, and its genome is rich in transposable elements (Mikkelsen et al., 2007). The expansion of CTCF-binding events numbers in the hundreds, and the CTCF-bound MIR elements in opossum contain only M1 motifs, with no evidence of associated M2 motifs (Figure 4D).

Perhaps surprisingly, we found no evidence of enrichment of CTCF-binding events within species-specific repeats in human or macaque, nor did we discover recent activity of retrotransposon expansion of CTCF within these two species.

Nevertheless, CTCF binding has expanded via retrotransposition in multiple, independent, diverse mammalian lineages; therefore, this mechanism of regulatory evolution is a profoundly ancient strategy that must predate the mammalian radiation.

### Molecular Paleontology of Fossilized, Repeat-Driven CTCF Expansions

If the repeat-driven mechanisms currently active in creating functional CTCF-binding events were also active in the common mammalian ancestor, then ancestral expansions would eventually become shared binding events in descendant species, such as our study species. Such a model would explain both the origin of shared CTCF-binding events as well as lineage-specific expansions via the same mechanism.

This hypothesis predicts that fossilized repeat sequences from ancient repeat expansions will be found around loci bound by CTCF in multiple mammals. However, tens of millions of years of evolution would likely have altered the genetic sequences surrounding the bound CTCF motif and so eliminated systematic evidence of associated repeat elements that could be obtained using a purely computational approach.

**Figure 5:**
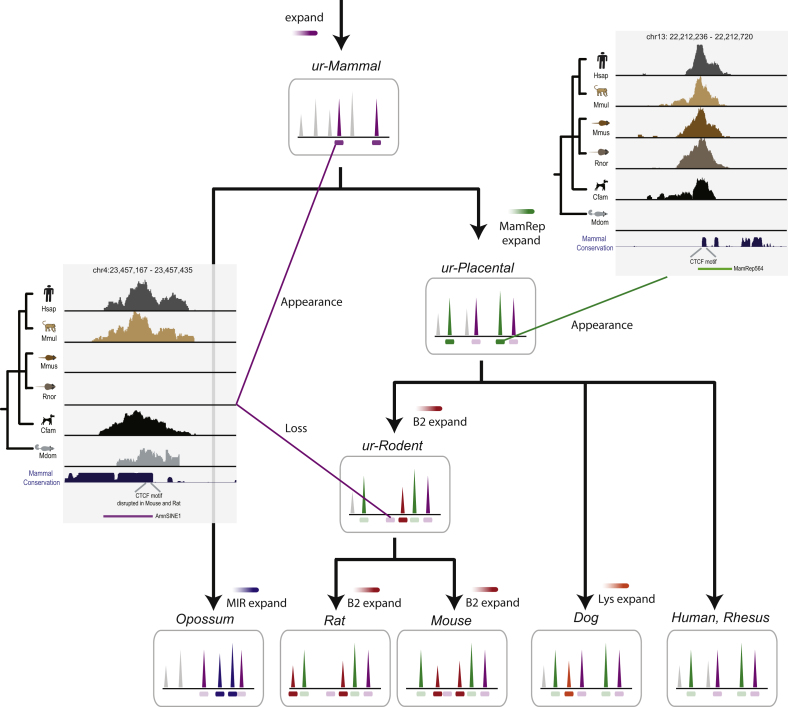
Intermittent Repeat Expansions Can Lead to Conserved, Lineage-Specific, and Species-Specific CTCF Binding in Mammals A CTCF-binding site found within an ancient transposon shows conserved binding in placental and nonplacental mammals (left data inset) and must have been present in the mammalian ancestor (ur-Mammal). In contrast, a CTCF-binding site generated in the eutherian ancestor (ur-Placental) shows conserved binding across placental mammals but is absent in marsupials (right data inset). More recent CTCF-binding expansions lead to increasingly lineage- and species-specific CTCF binding. For example, the expansions of B2 repeats in the mouse and rat ancestor (ur-Rodent) created CTCF binding that is highly shared between mouse and rat, whereas the continued B2 expansions along both lineages also generated species-specific CTCF-binding sites (see Figure 4C). See also Table S3.

Taking a more targeted approach that exploited our six species' in vivo experimental data, we looked for evidence in any genome of repeat element survival within the set of partially—or fully—shared CTCF-binding events. We found just over 100 CTCF-binding events (Table S3), often very deeply conserved, which had fossilized repeat sequences surrounding them in one or more of the mammals we profiled (Figure 5).

In Figure 5, we show two representative examples of candidate CTCF-binding events carried by ancestral repeats. First, on HsChr13, we identified a partially, though deeply, shared CTCF-binding event located within an ancient amniote SINE element (Figure 5) (Hirakawa et al., 2009). Interestingly, this specific binding event was lost along the rodent lineage due to a motif disruption in the common rat-mouse ancestor. Second, on HsChr4, a highly conserved CTCF-binding event is found associated with a copy of mammalian repeat MamRep564, which is shared among all placentals but appears to have arisen subsequent to the placental-marsupial split.

These examples, along with the larger set of partially preserved repetitive elements associated with shared CTCF binding (Table S3), lend support to a model wherein repeat-carriage of CTCF binding created highly conserved CTCF-binding events throughout mammalian and most likely vertebrate evolution.

### Newly Created, Repeat-Driven CTCF Expansion Events Demarcate Chromatin and Gene Expression Domains

**Figure 6:**
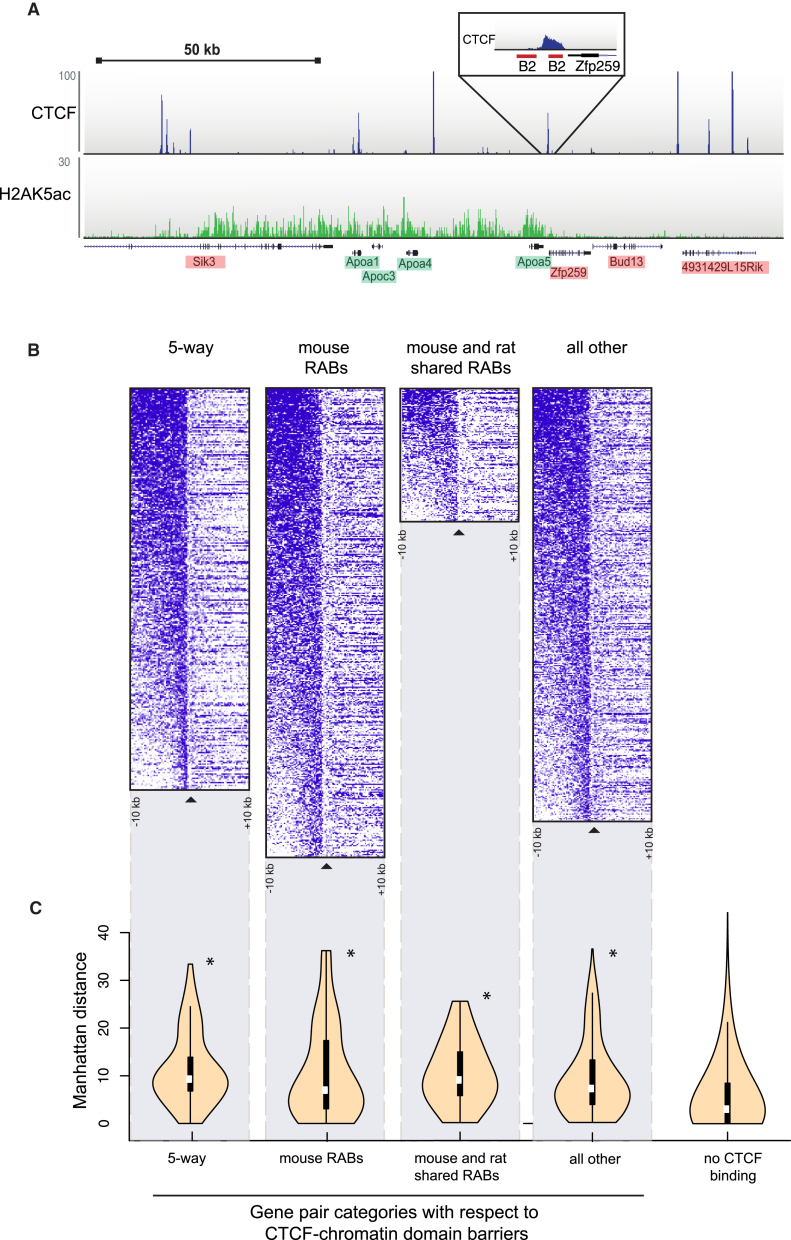
Chromatin Boundaries Separated by Repeat-Associated CTCF Binding in Rodents (A) A B2-associated CTCF-binding event separates the ApoA cluster from downstream genes on mouse chromosome 9 (top blue track). Active transcription is reflected both by H2AK5ac occupancy in mouse liver (bottom green track) and in direct sequencing of mouse liver mRNA by gene name shading (red is silent; green is active) (Mortazavi et al., 2008). (B) Heat map representation of H2AK5ac chromatin domains flanked by CTCF binding that is shared between all five species (five-way), mouse unique and repeat-associated (mouse RABs), repeat-associated and shared between mouse and rat (mouse and rat shared RABs), and not within the previous categories (all other). (C) Violin plots represent gene expression differences (Manhattan distances) between H2AK5ac and CTCF defined chromatin domains for different gene pair categories. See also Figure S6.

To assess the functional impact of SINE-driven CTCF-binding events on chromatin, we explored CTCF's known role as a barrier element that divides chromatin domains (Cuddapah et al., 2009; Xie et al., 2007). We reasoned that genomic locations where CTCF plays a functional role in separating chromatin domains would show distinct changes in histone modifications to either side of the CTCF-binding event. We therefore profiled the genome-wide location of histone 2A lysine 5 acetylation (H2AK5ac) (Cuddapah et al., 2009) and directly compared these data with matched CTCF occupancy data. This analysis identified hundreds of regions of abrupt changes in active chromatin demarcated by CTCF binding, consistent with CTCF's role as a barrier element and representing almost 5% of CTCF-binding events. Negative controls, such as unrelated TFs and random regions, showed only background level association with H2AK5ac in liver (Figure S6). In mouse, approximately 25% of CTCF chromatin boundaries were found to be associated with repetitive element expansion. For example, in mouse a CTCF-binding event found within a B2 SINE represented the boundary between the highly transcribed, liver-specific *ApoA* cluster of genes and the neighboring genes downstream on chromosome 9 (Figure 6A).

We asked whether newly expanded CTCF-binding events function as chromatin barriers as often as the five-way shared CTCF-binding events that predate the placental mammalian expansion. We exploited the recent expansion of B2 elements that have introduced thousands of novel, lineage-specific CTCF-binding events to the mouse genome. We categorized mouse CTCF binding by whether it was (1) conserved in all five placental mammals, (2) present in a mouse-specific SINE repeat, (3) present in a rodent-shared SINE repeat, and (4) all other binding events, as well as adding (5) random genomic regions as controls (Figure 6B). CTCF-binding events demarcate active and inactive chromatin at a similar frequency, regardless of whether the CTCF-binding events are shared between the eutherian mammals, rodent B2 associated, or mouse-specific B2 associated. Likewise, all CTCF boundaries are capable of demarcating transcriptionally active and inactive chromatin. Genes divided by CTCF-demarcated chromatin domains had higher transcriptional divergence (Figure 6C). In addition, we did not observe specific motif features associated with CTCF barrier elements, as CTCF binding generated from B2 transposons is equally likely to form CTCF barrier elements, as is non-repeat-associated CTCF binding.

**Figure 7:**
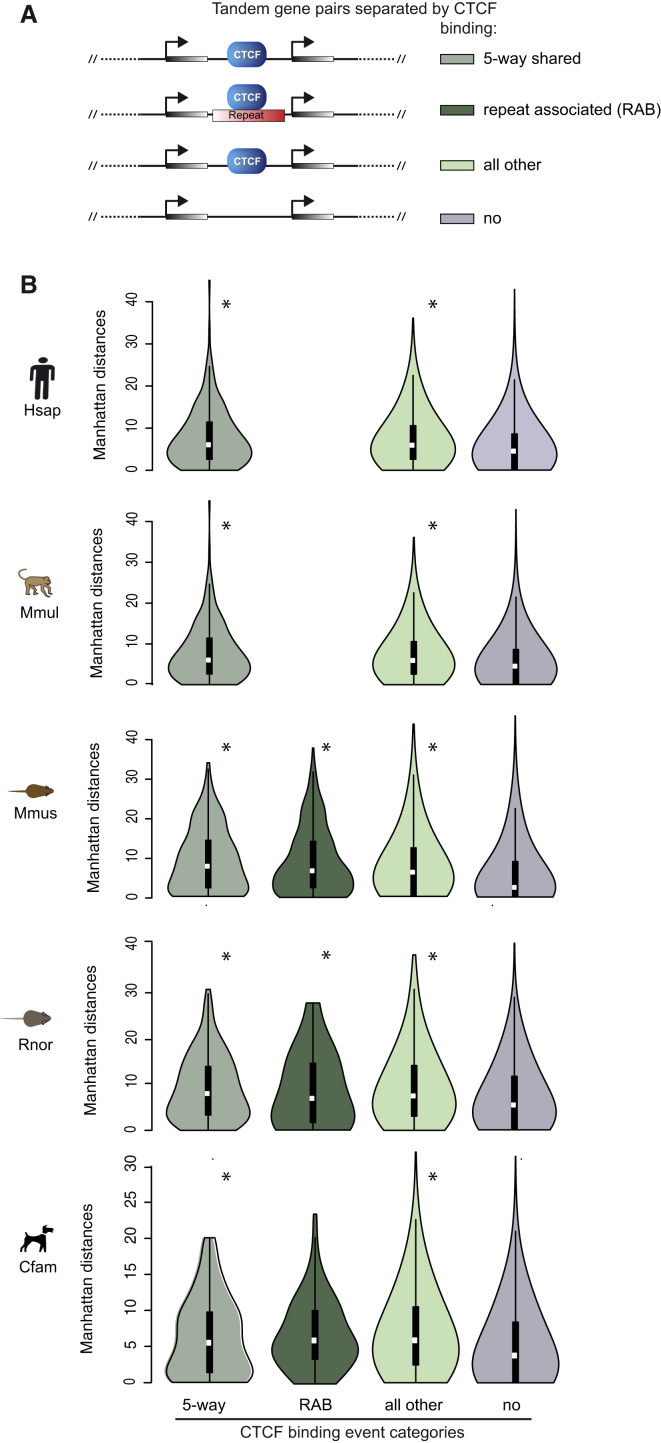
Tandem Gene Pairs Separated by CTCF Differ More in Their Expression than Gene Pairs that Are Not Separated by CTCF (A) Exemplified tandem gene pairs that are separated by CTCF binding or not separated by CTCF (no). The CTCF-separated tandem gene pairs are further distinguished into the following three groups: (1) shared between the five mammals shown in (B) (five-way shared), (2) associated with lineage-specific repeats (repeat-associated, RAB), (3) all other CTCF-separated gene pairs (all other). (B) Violin plots represent gene expression difference distributions (Manhattan distance) per tandem gene pair group as explained in (A). Stars (^∗^) indicate p values compared to the no CTCF binding category that are smaller than 0.001 (wilcoxon rank-sum test).

To further assess the functional impact of SINE-driven CTCF-binding events on transcription and gene expression, we explored whether CTCF can act as a transcriptional insulator between tandem genes (Figure 7A). Tandem genes are transcribed by RNA polymerase in the same direction and have been shown to have more coherence in their relative gene expression than non-tandem-organized genes (Caron et al., 2001; Lercher et al., 2002). We collected mRNA sequencing data in livers of all studied species, identified the subset of tandem genes divided by at least one CTCF-binding event in each species, and further subdivided this set by whether the CTCF-binding event was shared, repeat associated, or neither. In all species, we observed statistically significant increases in gene expression differences between tandem genes divided by CTCF (Figure 7B). We did not find any significant effects of the presence or absence of M2 on transcriptional insulation (data not shown). Indeed, repeat-associated CTCF-binding events in mouse, rat, and dog serve to transcriptionally separate members of tandem gene pairs.

Our data thus indicate that newly arisen CTCF-binding events in multiple mammalian species functionally demarcate chromatin domains and influence transcription at a similar frequency as do ultra-conserved CTCF-binding events.

## Discussion

Understanding the structure, function, and origins of the genome is fundamental to understanding the mechanisms of mammalian evolution. By assaying CTCF binding in matched tissues of six diverse mammals, we generated high-resolution in vivo maps of CTCF evolution. This uncovered over 100,000 previously unidentified CTCF-binding events in multiple species. Our data reveal a highly conserved 33/34 bp motif consisting of a two-part profile for CTCF binding, confirm that CTCF binding is remarkably conserved compared to other TFs, and demonstrate that CTCF has a core set of over 5,000 bound regions shared among five representative placental mammals. Word-level analysis of the binding events revealed a conserved motif hierarchy, and that new CTCF-binding events are born in highly diverse mammalian lineages via the expansion of repetitive elements. Many of these newborn CTCF-binding locations function as barriers to both chromatin and gene expression. Finally, we provide compelling evidence that the same process that currently drives lineage-specific expansion of CTCF-binding events in diverse mammals ancestrally generated the core set of strong, deeply conserved CTCF-binding events.

### Insights from an Expanded CTCF-Binding Motif

A larger motif for CTCF binding explains ambiguous results from prior studies (see also Figure S2), which suggested that the regions bound by CTCF are larger than 20 bp. For instance, the 40 bp DNase I footprint at the APP gene promoter sequence (Quitschke et al., 2000) contains the full motif we have identified (Figure 2E). Earlier work described CTCF as a multivalent TF that binds to two different DNA sequences in human (CTCF human fragment A) and chicken (CTCF fragment V) (Filippova et al., 1996). Our results explain that chicken fragment V contains the previously known 20-mer CTCF motif (Figure 2F) and is thus readily bound in vitro by zinc fingers 2 to 7; in contrast, the human fragment A contains the full 33 bp motif and thus requires the additional four C-terminal zinc fingers to be bound in vitro. A 3 bp mutation within the critical DNA bases of the M2 motif abolished CTCF binding (Figures 2F and S2) (Filippova et al., 1996). The existence of a set of CTCF-binding events that require the M1 and M2 motifs over the sole presence of the constitutive M1 motif can also help explain the exceptional conservation of CTCF's 11 zinc finger DNA-binding domain (Klenova et al., 1993; Moon et al., 2005). From the interaction of the C-terminal zinc fingers with the M2 motif, we can also deduce CTCF's orientation relative to the binding sequence. The expanded CTCF-binding motif helps explain previous, somewhat conflicting results and supports recent reports describing a preferred orientation of CTCF binding relative to its target sequence (Quitschke et al., 2000; Renda et al., 2007).

### The Genetic Architecture and Regulatory Implications of CTCF-Binding Conservation

The structural and regulatory organization of the mammalian genome is fundamentally dependent on CTCF (Phillips and Corces, 2009). Prior studies have revealed in the context of the rapid divergence of tissue-specific TF binding that CTCF binding is comparatively well conserved between human and mouse cell lines (Bourque et al., 2008; Kunarso et al., 2010). Reflecting the organizational role of CTCF, one of the few hundred CTCF-bound regions reported as shared among human, mouse, and chicken cells has been shown to serve as a genomic barrier to redirect EVI5 intron-located enhancers to regulate the distal GFI1 gene (Martin et al., 2011).

Our global data extend these studies, exploring CTCF-binding evolution in matched tissues from six mammals. This comparison revealed that conserved CTCF binding often shows a number of specific features, including the following: (1) tissue invariance, (2) a specific and conserved motif-word composition, (3) high ChIP enrichment, and (4) high-affinity protein-DNA interactions, as shown by strong resistance to RNAi-mediated knockdown. In contrast, most tissue-specific mammalian TFs not only evolve rapidly in their genomic binding but also differ from CTCF in most other features as well (Kunarso et al., 2010; Mikkelsen et al., 2010; Odom et al., 2007; Schmidt et al., 2010b). The conserved set of CTCF-binding events, therefore, represent an organizational pattern present in all mammalian cells, regardless of the developmental stage and tissue, and delineate chromatin structures required for conserved genome functions (as explored at one genomic locus; Martin et al., 2011).

### Repeat-Driven Expansions of CTCF Binding Are an Ancient and Ongoing Source of Genome and Regulatory Evolution

Due to CTCF's long, high information content motif, new CTCF-binding events are dramatically less likely to be generated by random mutations than binding events for TFs targeting much shorter motifs. However, the copy and paste mechanism of transposable elements is blind to size. Therefore, once a CTCF motif has been acquired by a transposon, it can spread across the genome by generating carbon copies of the originally gained motif sequence. Our experiments revealed that repeat-associated binding expansion carried functional CTCF-binding events throughout the muridae, canidae, and didelphidae genomes, suggesting that most mammalian lineages are subject to similar CTCF expansions. Interestingly (and perhaps surprisingly), our data in human and macaque show no evidence of such events. It is possible, however, that primate lineages that we have not yet studied have indeed been subject to repeat-driven expansion of CTCF binding, as other primate SINEs such as Alu elements have been active recently.

Expansions via transposable elements are increasingly recognized as a general mechanism for the generation of new binding sites of TFs with complex binding motifs (Johnson et al., 2006, 2009; Mortazavi et al., 2006). In addition, recent reports provide evidence that transposable elements contain sequences for larger regulatory assemblies that restructure tissue-specific transcriptomes (Lynch et al., 2011; Kunarso et al., 2010). For example, many binding events of the neuronal repressor NRSF/REST have been generated across vertebrate genomes by means of lineage-specific transposons (Johnson et al., 2006, 2009; Mortazavi et al., 2006), and the composite OCT-SOX motif has been expanded in humans (Kunarso et al., 2010). Similar expansions of retrotransposons that carry CTCF binding might, in fact, have an evolutionary advantage over those that do not: it has been shown that CTCF binding can prevent DNA methylation and the establishment of repressive chromatin modifications (Lee et al., 2010; Rand et al., 2004). Consequently, CTCF binding might provide transposable elements with a survival strategy, by protecting them against repressive chromatin and DNA modifications. Alternatively, CTCF and similar factors may have been part of genomic defense strategies against specific transposable element invasions.

Taken together, our data support a model in which lineage-specific repeat expansions have been propelling distinct CTCF motif-words and their associated binding events across the genome many times throughout mammalian evolution (Figure 7). The same mechanisms creating lineage-specific CTCF binding in extant species are almost certainly responsible for creating the ancient CTCF events found across all mammals. Despite the gradual divergence of genetic sequences surrounding the core CTCF sequence motif, we found evidence that multiple repeat sequences have carried CTCF binding in common ancestors. Indeed, deliberate molecular paleontology across our data revealed over a hundred such repeat fossils associated with conserved CTCF binding.

How repeat elements can globally contribute toward organismal phenotypes, from tissue-specific gene expression (Kunarso et al., 2010) to coat color (Blewitt et al., 2006) to lactation (Lynch et al., 2011), has only begun to be explored. Here, we have described how mammalian repeat elements are a major mechanism by which CTCF binding is born, thus revealing how complex eukaryotic regulatory structures and the repetitive sequences they control can collaborate to drive genome evolution.

## Experimental Procedures

We performed chromatin immunoprecipitation experiments followed by high-throughput sequencing (ChIP-seq) (Schmidt et al., 2009) using liver material isolated from six mammalian species: human (Hsap; primate), macaque (Mmul; primate), dog (Cfam; carnivora), mouse (Mmus; rodent), rat (Rnor; rodent), and short-tailed opossum (Mdom; didelphimorphia). For each ChIP experiment, at least two independent biological replicates from different animals, generally two young adult males, were performed (see Extended Experimental Procedures). ChIP-seq experiments were performed as recently described (Schmidt et al., 2009), and most interspecies analyses were performed as previously reported (Schmidt et al., 2010b).

**Figure S5:**
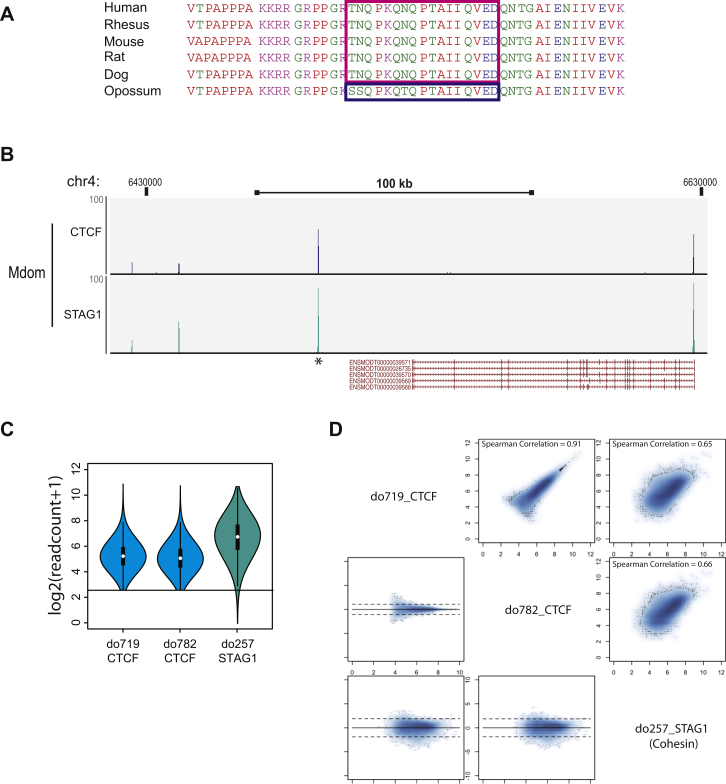
Custom Opossum CTCF Antibody Design and Validation, Related to Experimental Procedures (A) Alignment of parts of CTCF's protein sequences in multiple mammals. The peptides used to generate the commercial (human, rhesus, mouse, rat, dog) and custom (opossum) antibodies are highlighted. (B) Wiggle tracks of CTCF and cohesin (STAG1/SA1) binding in opossum liver around the APP1 gene. The binding event highlighted with a star is in the orthologous location of the human binding event used for DNase I footprinting (Quitschke et al., 2000). (C) Violin plots of raw read counts in opossum CTCF binding events for both replicates and cohesin validating that most opossum CTCF-binding events show strong cohesin enrichment. (D) Scatter and Bland-Altman plots comparing the opossum CTCF to the opossum cohesin replicates. Spearman correlations are indicated.

The CTCF antibody 07-729 (Milipore) was used for all experiments except the opossum ones, which were performed using a custom antibody as described and validated in Figure S5. The custom opossum CTCF antibody is available upon request. The STAG1 antibody used for validation of the opossum results and the H2AK5ac antibody were both purchased from abcam, ab4457 and ab1764, respectively.

Extended Experimental Procedures All scripts were written in Perl (http://www.perl.org), Python (http://www.python.org), R (http://www.r-project.org; R Development Core Team, 2008), or Bioconductor (http://www.bioconductor.org; Gentleman et al., 2004), using the packages GenomicRanges, ShortRead, Sgenome, Biostrings, gtools, and gplots.

**Source and Detail of Tissues**

We performed chromatin immunoprecipitation experiments followed by high-throughput sequencing (ChIP-seq) (Schmidt et al., 2009) using liver material isolated from six mammalian species: human (Hsap; primate), macaque (Mmul; primate), dog (Cfam; carnivora), mouse (Mmus; rodent), rat (Rnor; rodent), and short-tailed opossum (Mdom; didelphimorphia). For each ChIP experiment, at least two independent biological replicates from different animals were performed. The Cfam (2 adult males; 14 months of age), Rnor (2 adult males; 2.5 months of age), and Mmul (2 adult males; 18 years and at least 18 years of age, one adult female 18 years of age) livers used in this study were obtained from commercial sources. Healthy human hepatocytes (Hsap, 1 male; unknown age, 1 female, unknown age) were obtained from the Liver Tissue Distribution Program (NIDDK Contract #N01-DK-9-2310) at the University of Pittsburgh and the Addenbrooke's Hospital at the University of Cambridge under license number 08-H0308-117 “Liver specific transcriptional regulation.” Mdom livers (2 adult males; 17 months of age) were obtained from the University of Glasgow, UK. Mmus (two adult C57BL6/J males, 2.5 months of age) were obtained from the CRI under Home Office license PPL 80/2197.

**Derivation of a Monodelphis-Specific CTCF Antibody**

Because the epitope used to create the placental-specific CTCF antibody is not conserved in marsupials (Figure S5A), we generated a custom antibody against the opossum CTCF protein sequence. We confirmed this antibody's specificity by showing high correlation of occupancy between CTCF and the cohesin subunit Stag1 (Spearman correlation = 0.62, Figures S5B–S5D). Stag1 is highly conserved among mammals and has been shown to extensively colocalize with CTCF (Rubio et al., 2008; Schmidt et al., 2010a). See Figure S5 for validation details.

**Sequence Alignment and Peak Calling**

**Figure S6:**
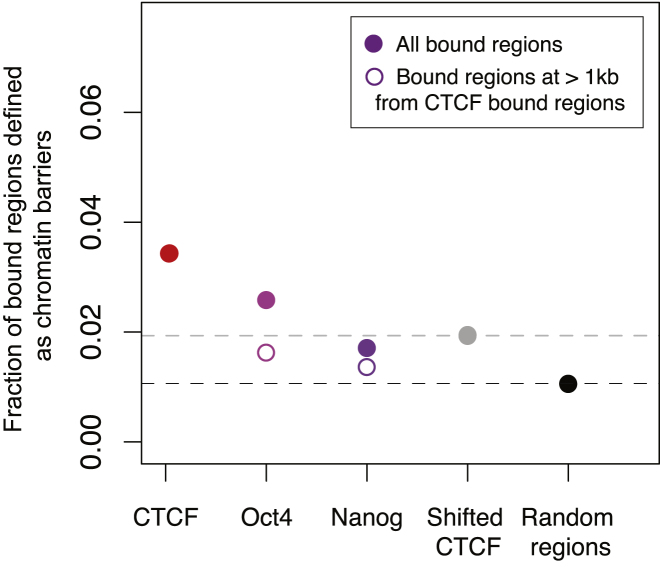
Colocalization of TFs with Chromatin Barriers, Related to Figure 5 The fractions of regions bound by CTCF in mouse liver as well as Oct4 and Nanog in mouse ESCs (Marson et al., 2008) that are found to be at mouse liver H2AK5ac domain boundaries are shown. Open circles indicate Oct4 and Nanog binding events that are more than 1 kb away from a CTCF-binding event. As random controls we shifted CTCF binding randomly (Shifted CTCF) and selected a set of random genomic regions (Random regions).

ChIP and input sequencing reads were trimmed to 36 bp and aligned using Bowtie 0.11.2 with the parameters “-n 2–best -m 3” to the following genome assemblies: mouse NCBI m37, rat RGSC3.4, human GRCh37, rhesus macaque Mmul_1, dog CanFam2.0, and opossum MonDom5 (Table S1A). All sequence, genome annotations, and comparative genomics data were taken from Ensembl releases 57 and 60 (http://mar2010.archive.ensembl.org/; http://nov2010.archive.ensembl.org/). After alignment, biological replicates were merged and binding events were detected with SWEMBL (http://www.ebi.ac.uk/∼swilder/SWEMBL/), with the parameters “-R 0.005 -i -S.” To assess the variation between individual replicates, we counted the individual tags that map to peak regions and calculated Spearman correlation coefficients between samples (Table S2B). The same analysis was performed between the opossum CTCF replicates and the opossum cohesin (STAG1) experiment. The data were further quantile normalized for the scatter and Bland–Altman plots in Figure S6 to correct for differences in sequencing depth between replicates.

**Interspecies Analysis**

We performed all our interspecies comparisons based on the 13-way amniota vertebrate (PECAN), as well as 11-way eutherian mammals (EPO) multiple sequence alignment (MSA) available in Ensembl Compara. Binding events discovered by SWEMBL, PWM matches for the canonical CTCF motif, or repeat elements were projected onto all study species using the MSA through the Ensembl Compara Application Programming Inteface (API). We restricted the evolutionary analysis to regions of the genome included in the MSA. Each of the six study species was used as anchor species, and then the region of interest projected onto the five other species. For determining the degree of commonality between the species, projections were then overlapped with CTCF-binding events (or the detected PWM matches for motifs, respectively the RepeatMasker annotated repetitive regions for repeat elements) and sharing categories determined. Overlap numbers differ by tens of bound regions depending on which species is used for anchoring. The percentage overlap numbers reported in Figure 1A are averages between the two analyses directions (e.g., shared human-dog sites from human and dog perspective). The five-way overlap numbers shown in Figure 1B are human centric. Conservation numbers reported in the manuscript are based on the PECAN alignments; we note that absolute EPO numbers are slightly higher (e.g., 6,946 five-way shared bound regions in human out of a total of 50,482 human CTCF-bound regions included in the EPO alignment), as a higher fraction of the respective genomes are contained in the MSA. However, the relative conserved fractions are similar to the PECAN results: 15% (PECAN) respectively 14% (EPO) of human-bound regions are classified as five-way shared.

**Motif Conservation Analysis, Related to Figure 1**

We used the human TFs USF1, SRF, EGR1, ELK4, and GATA2 (available as wgEncodeHaibTfbs broadPeak tracks H1hescUsf1Pcr1xPkRep1/2, H1hescSrfPcr1xPkRep1/2, K562Gata2sc267Pcr1xPkRep1/2, K562Egr1V0416101PkRep1/2, H1hescEgr1V0416102PkRep1/2 and wgEncodeSydhTfbs narrowPeak tracks HuvecGata2UcdPk and Helas3Elk4UcdP), probed in the ENCODE project and used in accordance with the ENCODE Data Release Policy, published CEBPA and HNF4A data (Schmidt et al., 2010b), as well as NRSF/REST binding data in human livers. We employed GERP scores (Cooper et al., 2005) calculated over EPO space (Ensembl release 60) as a measure of evolutionary conservation. We performed de novo motif discovery on 50 bp located in the center of the 2,000 top and middle TF-bound regions with MEME (parameters “-nmotifs 5 -minsites 100 -minw 8 -maxw 25 -revcomp -maxsize 500000 -dna”) to obtain PWMs for all factors. We subsequently scanned all bound regions with the corresponding position weight matrix (PWM) and the cutoff −10, determining bound motif instances. We then calculated the median GERP score per bound motif region and plotted the median values against the motif information content and length. We tested the significance of the correlation between motif length/IC and median motif GERP scores in R using the function “cor.test” (method = “spearman”) for (a) all TFs: length versus GERP rho = 0.70, p value = 0.03; IC versus GERP Spearman's rho = 0.45, p value = 0.23 and (b) the seven TFs remaining after exclusion of CTCF and NRSF/REST: length versus GERP Spearman's rho = 0.34, p value = 0.42; IC versus GERP rho = 0, p value = 1.

**Motif Discovery and Analysis**

Motif discovery was conducted with NestedMica using the parameters “-minLength 5 -maxLength 30 -numMotifs 6” and a fourth order background model trained on mammalian regulatory regions data. Discovered motifs were confirmed using MEME, with the options “-nmotifs 5 -minsites 100 -minw 6 -maxw 25 -revcomp -maxsize 500000 -dna.” We selected the top 1,000 peaks ordered by SWEMBL score and used 25 bp up- and downstream of the peak summit as input to motif discovery. As the obtained top motifs were virtually identical in all studied species, we merged them into a single PWM that we used in further motif analysis steps. We refer to this motif as “M1.” Motif discovery revealed a second motif, present again in all species that we refer to as “M2.” In order to test the relationship between the two motifs, we calculated the distances between their centers and determined a preferred spacing of 20 and 21 bp. We calculated the background spacing by randomly choosing the same number of M1 motifs as found in bound instances and looking for the closest downstream M2 (12 to 42 bp spacing). We plotted the median value obtained after 100 repetitions (shown for human in Figure 2D).

**CTCF Motif Sequence Conservation Analysis, Related to Figure 2D**

We calculate sequence conservation using GERP scores (Cooper et al., 2005) based on the binding sites in the human genome and created from the PECAN alignments used (Ensembl release 57). We plotted the observed/expected GERP profile around the canonical CTCF motif for placental-shared sites (11111) that had both M1 and M2 (20 bp spacing) at detectable levels (cutoff −15). As a second method, we used base substitutions. We calculated the frequency of unchanged bases at five-way shared sites that had an M2 motif at a spacing of 20 bp in human (based again on PECAN alignments) and plotted them as a PWM.

**Motif-Word Analysis**

Individual motif instances obtained by scanning the genomes with canonical CTCF PWM were collected as DNA words (14-mers). We defined the set of bound words as the union of words falling inside bound regions in our study species. We also counted the background occurrence of these words, by looking at all PWM matches across each genome, irrespective of CTCF binding. We then plotted the frequency of the words (occurrence in bound regions divided by occurrence in genome) that were bound over five times in any species as a heatmap, sorted by the human column and hierarchically clustered (column-wise, using Spearman rank correlation). We grouped the sorted words into 25 bins and calculated the fraction of five-way bound regions containing 14-mers present in a particular group, as well as the average SWEMBL score per group. As a control, we performed the analogous analysis for HNF4A, using the three species data available from Schmidt et al. (2010b) and a PWM match cutoff of −10. For CTCF, we counted individual occurrences of all motif-words in the studied species and divided by a normalization factor, proportional to the total number of bound bases in a certain species, obtaining a normalized occurrence (nocc) measure for each word and species: nocc_i,j_ = occ_i,j_/factor, where occ is the word count, i is the word number, j is the species number, and factor is defined as the total bound bases divided by 1,000,000. We then used the normalized word occurrence values to define species-specific words.

**Species-Specific Word Analysis**

Species-specific words were chosen as ln(nocc(S))/nocc(R)) > 2 and nocc(S) > 8 where S = species of interest (one species for dog and opposum, two species for the rodent and primate lineage) and R = other species (5 studied species excluding S). For example, to define dog specific motif-words, all words with (ln(occ(dog))/nocc(primates+rodents)) > 2 and nocc(dog) > 8 were considered. We tested the association of all detected word sets with repeat elements—as present in the UCSC RepeatMasker tracks—by calculating the fraction of species-specific words with different repeat elements families, classes and names, starting at the family level and progressing toward individual repeat assessments. We found significant (one sided Fisher's exact test; p value < 10^−40^) association of rodent-specific words with individual members of B2 elements in mouse and rat, of Lys family repeats in dogs and MIR family elements in opossum. We did not detect any primate word sets significantly associated with repeats. We show the log_2_ normalized occurrence of species-specific words as a column-clustered heatmap in Figure 4A. We next tested genome-wide association of the three repeat classes and their subtypes with CTCF-binding events (binomial test, background probability estimated using median of randomized binding event-sized regions located in genome areas delimited by CTCF-binding events) and calculated the number of repeat elements overlapping a bound CTCF motif hit. We next aligned all bound CTCF motifs located inside these repeats and derived species-specific PWMs that essentially reproduced the observed species-specific motif-words. For all repeats, we determined the number of bound instances, as well as the presence and spacing of M1 and M2.

**Chromatin Boundaries Analysis**

For the chromatin boundary analysis, we determined regions of H2AK5ac enrichment compared to input by using MACS (Zhang et al., 2008) with the parameters “–nomodel–shiftsize = 100–bw = 100–tsize = 36–mfold = 20” and defined CTCF barrier sites as described previously (Cuddapah et al., 2009) - “a CTCF binding site, denoted by genomic coordinate x, is defined as a barrier site relative to a H2AK5ac domain d of length l, only if the distance between x and the domain boundary is at most the smaller of l/10 and 1000 bp.” For four different CTCF binding events categories—five-way shared, mouse-specific RAB, mouse-rat shared RAB and all other bound regions—we plotted log_2_(extended-read counts) 20 kb around the CTCF peak summit, orienting the regions on the H2AK5ac signal. We used three distinct controls to obtain a background expectation of barrier numbers: (1) regions bound by the TFs Oct4 and Nanog in ESCs available from (Marson et al., 2008); (2) CTCF-bound regions shifted by a random distance between 2 and 100 kb and (3) randomly distributed regions of the same size and number as CTCF-binding events. We show the fraction of CTCF, as well as negative control regions classified as barriers in Figure S5. For Oct4 and Nanog both unfiltered numbers and fractions after removal of bound regions located at <1 kb of a CTCF-binding event are shown.

**Tandem Gene Pair Expression Analysis**

We used RNA-seq data in the five eutherian to test CTCF's insulator activity by looking at genes arranged in tandem in the genome (TSS of gene 1 will be at <10 kb of TES of gene 2, genes are nonoverlapping and on the same strand). We compared the Manhattan distances of log_2_(transcript estimates) for tandem genes separated by five-way shared, repeat-associated and “regular” CTCF-binding sites with tandem genes not separated by any CTCF-binding sites by using a Wilcoxon singed-rank test. We also compared Manhattan distances of genes (nonoverlapping, distance < 10 kb between gene bodies) separated by a CTCF chromatin barrier (five-way, mouse-specific, mouse-rat shared, and “regular”) to genes with no CTCF binding between them, using a Wilcoxon singed-rank test.

**CTCF Knockdown Analysis**

We used publicly available CTCF-binding data before and after RNAi-mediated CTCF knockdown in human MCF-7 cells from Schmidt et al. (2010a). We first overlapped CTCF-bound regions in human liver with those in MCF-7 cells, obtaining a common set of bound locations. We then categorized these common regions according to their conservation in liver tissue—five-way shared versus human-specific—and calculated the RNAi-resistant fraction in both categories, as displayed in Figure 1D. We included all relevant numbers in Figure S1. We show median normalized ChIP signals ((sum(ChIP)+1)/(sum(Input)+1))∗n, where n is total(ChIP)/total(Input)) before and after CTCF knockdown 1 kb around the CTCF summit at five-way shared and human-only binding events in Figure S1.

**Annotation Analysis**

We submitted the human coordinates of the five-way shared CTCF bound regions to GREAT version 1.8 (McLean et al., 2010) using default parameters (Basal+extension: 5000 bp upstream, 1000 bp downstream, 1,000,000 bp max extension) and included significant associations for “GO Terms Biological Process,” as well as “MGI Phenotypes” in Table S2. To test the association of B2 embedded, respectively mouse-specific CTCF bound regions with mouse-specific genes, we submitted the mouse coordinates of different types of CTCF-bound regions to GREAT (using again default parameters) and obtained sets of genes proximal to (1) B2-associated, (2) non-B2-associated, (3) mouse-specific within alignments, (4) mouse-specific outside alignments, and (5) five-way shared CTCF-binding events. We then used Ensembl 60 orthology relationships to determine mouse and rodent-specific genes, and report the relative fraction of such genes for the different binding site categories in Figure S4D.

**Related to Figure 1**

The CTCF-binding tracks shown in Figure 1C exhibit complexities that may not be expected from such a direct experiment. The number of tags expected to map to a binding event is not only dependent on the binding level at a particular site, but also the composition of the total binding event population that is being sampled. As intended, the RNAi treatment changes the binding event population and therefore the expected number of reads of a particular binding event might even increase in the knockdown experiment relative to the control experiment.

Mikkelsen et al. (2007) proposed a simple model for the number of sequence reads in a chromatin feature:

“Suppose that the genome is divided into *N* non-overlapping bins of fixed size, that a fraction *f* of these bins contain a particular chromatin feature and that one performs ChIP-Seq with an antibody that enriches the sequence in these bins by a factor of *e*. If one collects a total of R sequence reads, the number of reads in a bin should approximately follow a Poisson distribution with mean *eM* for bins containing the feature and *M* for the other bins, where *M* = *R/N*(*ef*+(1-*f*)).”

Thus, when the number of binding events decreases (*f)* (as would be expected in an RNAi experiment) the observed number of reads per chromatin feature (*eM*) will increase, when the enrichment factor e is unchanged for the remaining features. For example: Given the above formula and assuming that (1) we sequenced and aligned 20 million reads, (2) enrich for CTCF bound regions 30-fold, (3) bound regions are 500 bp in size, and (4) before RNAi we have CTCF bound to 1% of all 500 bp bins in the genome and after RNAi to half of those (0.5%), we would expect that the number of reads in the remaining population of CTCF binding events to follow some distribution with the mean of 75 before and 85 after CTCF knockdown and thus increase despite the RNAi condition.

This is consistent with the presented RNAi data. The observed increase in read coverage at some binding events is due to the decrease in enrichment or disappearance of other binding events. The interdependence of read counts of seemingly independent features in sequencing analysis is a general problem not only in ChIP-seq but also in RNA-seq and other applications that compare samples from different populations. Currently models are being developed to normalize for those effects (Garber et al., 2011; Robinson and Oshlack, 2010).

**Related to Figure 2**

The M2 motif identified within a subset of the CTCF binding events could be explained by (1) direct interaction of CTCF with M2 or (2) interaction of another molecule (protein or RNA) with M2, as suggested by Boyle et al. (2011). Here we further elaborate on the content of the figure, as well as previous seminal studies, that together indicate that CTCF's DNA-binding domain does directly interact with both M1 and M2.

The first observation that indicates direct interaction of CTCF with M2 comes from the spacing and orientation of M1 and M2 relative to each other. Both motifs are found in the same 5′ to 3′ orientation at only two significant spacings in all of the six species, resulting in a 33 and 34 bp two part motif, and ruling out potential genome sequence or assembly biases. This size motif is consistent with the fact that a DNA-binding protein with 11 zinc fingers should in principle target a 33 or 34 bp wide motif (Pavletich and Pabo, 1991). A traditional method to identify the DNA bases bound by a TF is DNase I footprinting. Quitschke and colleagues performed such an experiment using CTCF and the promoter sequence of the amyloid precursor protein gene (APP) (Quitschke et al., 2000), which is a known CTCF target (Vostrov and Quitschke, 1997). They used not only purified CTCF from HeLa cells, but also recombinant expressed and purified CTCF from *Pichia pastoris*. Their observations were consistent regardless of the origin of the purified CTCF protein, suggesting that their results were unlikely to be influenced by potential contaminations of the CTCF extracts. The observed footprint for CTCF comprised 40 bp (Figure 2E) (Quitschke et al., 2000). We performed motif analysis of the footprint's sequence and detected the presence of both M1 and M2. Furthermore it was shown that the protection of the M2 motif is lost when the most C-terminal zinc fingers of CTCF are deleted, suggesting direct interaction of these with the M2 motif (Quitschke et al., 2000). Interestingly, they observed a DNase I hypersensitive site within the CTCF footprint, which lies within the noninformative bases of the M1+M2 motif at position 23 and is lost in C-terminal deletions.

CTCF binds the orthologous sequence of the *APP* promoter in all our studied species. The full M1+M2 motif is found in all species out to opossum, corresponding to about 180 million years of evolution. The observed insertions, deletions, and single-base mutations do not disrupt the M1+M2 CTCF motif. For example, the 1 bp deletion in mouse and rat is within the noninformative bases of the M1+M2 motif and results in the second preferred spacing of 20 bp instead of the observed 21 bp in the other species—in effect, a very conservative alteration. The observed seven base pair insertion in syntenic dog and opossum sequences is outside of the M1+M2 motif.

All of these results suggest that CTCF's C-terminal zinc fingers are directly interacting with the M2 motif. Furthermore, the fact that the complete M1+M2 motif was maintained over 180 million years of evolution at a known functional target of CTCF underscores the importance of CTCF's interaction with M2 at the *APP* promoter.

The M2 motif is only detected in a subset of CTCF-binding events (Figure 2B), suggesting two modes of DNA-CTCF interaction. If CTCF indeed has two different modes of contact with DNA (M1 or M1+M2), we would expect a shift in the peak summit based on the presence and absence of M2. The expected shift is only about 7 bases and thus subtle but observable in human (Figure S2C) as well as the other five mammals (data not shown). This result would be best explained by direct interaction of CTCF with M1 in some cases, and M1 and M2 in the others.

Indeed it has been suggested that CTCF has variable modes of binding to DNA (Filippova et al., 1996). We investigated whether these seminal observations, which led the authors to describe CTCF as a “unique ‘multivalent’ transcriptional factor,” are consistent with our findings. Filippova and colleagues relied mainly on the analysis of two DNA sequences: the human fragment A and the chicken fragment V. The sequences of both fragments are shown in Figure S2E. Methylation interference analysis suggested that CTCF interacts with 30-40 bases of the fragment A and only about 20 bases of fragment V (Filippova et al., 1996). These results would be in agreement with our model of CTCF binding if fragment A contained a M1+M2 motif and fragment V contained only the previous CTCF motif M1. Indeed this is the case (Figure S2F). Further, mutation experiments showed that three bases of fragment A that correspond to high information content positions in M2 are critical for CTCF binding. This experiment was carried out with the in vitro translated DNA-binding domain of CTCF, thus ruling out artifacts from contaminations or the possibility that other parts of the full-length CTCF protein contact M2. Additionally, when bound to CTCF, fragment A protected more zinc fingers of CTCF from proteolytic degradation than fragment V, adding to the evidence that additional fingers are used to bind fragment A but not V. Indeed, further in vitro experiments performed by Filippova et al. showed that the most C-terminal finger of CTCF (zinc finger 11) is necessary to bind fragment A whereas fragment V is sufficiently bound by zinc fingers 1–7 only (Filippova et al., 1996).

Taking together the previous in vitro studies, as well as our genome-wide in vivo data, it is evident that CTCF interacts directly with M1+M2 using its zinc fingers. More precisely, the N-terminal fingers interact with M1 and the C-terminal fingers interact with M2 when present.

**Related to Figure S1**

To generate the heatmaps of the raw ChIP-seq data, the human binding events for CTCF that are within the multispecies alignment and have syntenic sequence on the human chromosome 21 in the Tc1 mouse were used as targets to center each window. Each window was divided into 100 bins of 100 bp in size. An enrichment value was assigned to each bin by counting the number of sequencing reads in that bin and subtracting the number of reads in the same bin of an input library. Each dataset was normalized to the same number of sequencing reads in the whole genome. Data were visualized with Treeview (Saldanha, 2004).

Panel C: Each sequencing read was lifted from its original genome sequence to the human genome using the UCSC liftOver tool (http://genome.ucsc.edu/cgi-bin/hgLiftOver) and the appropriate chain files. The minimum ratio of bases that must remap was set to 0.1 and multiple output regions were allowed.

Panel D: To compare CTCF conservation to other TFs, we used published CEBPA and HNF4A data (Schmidt et al., 2010b), in human, mouse and dog. We aligned the reads and determined bound regions with SWEMBL, following the same procedure as for CTCF. For each factor, we counted the binding events occurring in only one, exactly two and all three species for the four proteins (CTCF, CEBPA, and HNF4A) using the PECAN alignments in Ensembl release 57.

(E) We used CTCF-binding events available from the ENCODE project (ENCODE Project Consortium, 2011) to enquire the relationship between CTCF binding conservation and tissue specificity. We define a set of tissue-invariant sites as binding events present in the human ENCODE cell lines Gm19238, Gm19239, H1hesc, HeLA, Huvec, K562, MCF-7, and Progfib. We report the fraction of human-unique respectively five-way shared CTCF bound regions in liver overlapping ubiquitous binding events.

**Related to Figure S2**

To investigate a shift in the read distribution, we centered the CTCF motif on the 34 bp M1+M2 motif on the forward strand only and plotted the mean read number per bin divided by the maximum value for two categories: those with M1 only and those with both M1 and M2. To determine the number of bound events with motifs of different categories, we scanned the bound regions with both M1 and M2 using nmscan at a cutoff of −15, and counted (1) total number of peaks; (2) number of peaks with at least one M1 hit; (3) number of peaks with at least one M1 hit and an M2 hit at 12 bp to 42 bp half-site distance of M1; (4) number of peaks with at least one M1 hit and an M2 hit at 2 bp or 21 bp half-site distance of M1.To determine the degree of motif sharing, all human PWM scans with a score above −15 were projected onto the genomes of the other five study species using the PECAN-MSA, and the projections were overlapped with PWM hits in the respective species. The fraction of peaks with no, one, and over two motifs shared in two, three, four, and five species was then calculated.

**Related to Figure S3**

To confirm that the different repeat elements (mouse and rat, B3; dog, SINEC_Cf2 and SINEC_Cf3; and opossum, MAR1_Mdo) are indeed bound by CTCF in vivo, we plotted the mean read count per base centered around CTCF PWM matches inside bound repeats, as well as the repeat density (repeats per position) at the same genomic locations. To analyze the degree of commonality between mouse and rat binding sites associated with B3 elements, we compared the number of repeat and non-repeat-associated sites in the different sharing categories, such as mouse or rat specific (00100 and 00010), shared between mouse and rat (00110), or placental specific (11111), by using the PECAN MSA (Ensembl release 57), as described for the bound regions. The fraction of a category within the total repeat- or non-repeat sites is displayed. We estimated the age of individual repeat elements (for both bound and all repeats of a certain type) by dividing the number of substitutions from the consensus with the mutation rate estimated for mammalian species (2.2∗10^−9^ per base pair per year) (Kumar and Subramanian, 2002) and rodents respectively 4.5∗10^−9^ per base pair per year (Waterston et al., 2002).

## Accession Numbers

Data have been deposited under ArrayExpress accession numbers E-MTAB-437 and E-MTAB-424.

## Note Added in Proof

The presence of the full M1+M2 motif in a large subset of human CTCF-binding data was recently reported using a novel exonuclease digestion strategy.

Rhee, H.S., and Pugh, B.F. (2011). Comprehensive genome-wide protein-DNA interactions detected at single-nucleotide resolution. Cell *147*, 1408–1419.

## Supplemental Information

Table S1. Read Mapping Overview and Replicate Correlations, Related to Figure 1

Table S2. GREAT Analysis of Five-Way Shared CTCF Binding, Related to Figure 1 We used GREAT (McLean et al., 2010) to analyze the genome-wide distribution of five-way shared CTCF-binding events. Five-way shared CTCF binding in liver appears not to be enriched near liver-specific genes.

Table S3. Highly Conserved CTCF-Binding Sites Associated with Repetitive Elements, Related to Figure 7

Document S1. Article plus Supplemental Information
