# Waves of Retrotransposon Expansion Remodel Genome Organization and CTCF Binding in Multiple Mammalian Lineages

**DOI:** 10.1016/j.cell.2012.02.001

**Published:** 2012-02-17

**Authors:** Dominic Schmidt, Petra C. Schwalie, Michael D. Wilson, Benoit Ballester, Ângela Gonçalves, Claudia Kutter, Gordon D. Brown, Aileen Marshall, Paul Flicek, Duncan T. Odom

(Cell *148*, 335–348; January 20, 2012)

In the above article, the authors inadvertently listed an Array Express accession number as E-MTAB-438. The accession number has been corrected as E-MTAB-437 and is available with the article online.

