# Supplementary material for: Waves of Retrotransposon Expansion Remodel Genome Organization and CTCF Binding in Multiple Mammalian Lineages
Source: Cell. 2012 Jan 20;148(1-2):335–48. doi: 10.1016/j.cell.2011.11.058 (PMC3368268; doi:10.1016/j.cell.2011.11.058)
Supplement: Table S1. Read Mapping Overview and Replicate Correlations, Related to Figure 1 [file mmc1.pdf]

Table S1: Read mapping overview and replicate correlations, related to Figure 1
(A) Summary of total number of DNA sequence reads mapped using Bowtie to the following genome assemblies: mouse NCBI m37 (mm9); dog CanFam 2.0 (canFam2); human GRCh37 (hg19); opossum MonDom5 (monDom5); rat RGSC 3.4 (rn4); and rhesus macaque Mmul_1 (rheMac2).

(B) Spearman's rho was calculated between each CTCF ChIP-seq replicate to confirm reproducibility of CTCF ChIP-seq as described in the Extended Experimental Procedures.

Table S1A

CTCF	Aligned ChIP reads	Aligned Input reads
Hsap	26008297	24461768
Mmul	20101768	18913816
Mmus	25855946	17572031
Rnor	40716782	38323754
Cfam	22983440	24197289
Mdom	38176713	31915578
Total	173842946	155384236

Table S1B

Species	Sample 1	Sample 2	Spearman Correlation
Human	do506	do778	0.87
Rhesus	do45	do186	0.91
Rhesus	do45	do188	0.90
Rhesus	do186	do188	0.90
Mouse	do464	do781	0.95
Rat	do804	do807	0.98
Dog	do301	do780	0.79
Opossum	do782	do719	0.91
